# Systematic exploration of the underlying mechanism of gemcitabine resistance in pancreatic adenocarcinoma

**DOI:** 10.1002/1878-0261.13279

**Published:** 2022-07-22

**Authors:** Kaidong Liu, Yiding Geng, Linzhu Wang, Huanhuan Xu, Min Zou, Yawei Li, Zhangxiang Zhao, Tingting Chen, Fengyan Xu, Liang Sun, Shuliang Wu, Yunyan Gu

**Affiliations:** ^1^ Department of Systems Biology, College of Bioinformatics Science and Technology Harbin Medical University Harbin China; ^2^ Department of Human Anatomy, Key Laboratory of Preservation of Human Genetic Resources and Disease Control in China, Ministry of Education Harbin Medical University Harbin China; ^3^ The Sino‐Russian Medical Research Center of Jinan University, the Institute of Chronic Disease of Jinan University The First Affiliated Hospital of Jinan University Guangzhou China

**Keywords:** drug response signature, gemcitabine, immunity, pancreatic ductal adenocarcinoma, single cell

## Abstract

Resistance to gemcitabine is the main challenge of chemotherapy for pancreatic ductal adenocarcinoma (PDAC). Hence, the development of a response signature to gemcitabine is essential for precision therapy of PDAC. However, existing quantitative signatures of gemcitabine are susceptible to batch effects and variations in sequencing platforms. Therefore, based on within‐sample relative expression ordering of pairwise genes, we developed a transcriptome‐based gemcitabine signature consisting of 28 gene pairs (28‐GPS) that could predict response to gemcitabine for PDAC at the individual level. The 28‐GPS was superior to previous quantitative signatures in terms of classification accuracy and prognostic performance. Resistant samples classified by 28‐GPS showed poorer overall survival, higher genomic instability, lower immune infiltration, higher metabolic level and higher‐fidelity DNA damage repair compared with sensitive samples. In addition, we found that gemcitabine combined with phosphoinositide 3‐kinase (PI3K) inhibitor may be an alternative treatment strategy for PDAC. Single‐cell analysis revealed that cancer cells in the same PDAC sample showed both the characteristics of sensitivity and resistance to gemcitabine, and the activation of the TGFβ signalling pathway could promote progression of PDAC. In brief, 28‐GPS could robustly determine whether PDAC is resistant or sensitive to gemcitabine, and may be an auxiliary tool for clinical treatment.

## Graphical abstract

Using within‐sample relative expression ordering of pairwise genes, we developed a qualitative gemcitabine signature containing 28 gene pairs, named 28‐GPS. The 28‐GPS outperformed previous signatures in classification accuracy and prognostic performance for pancreatic ductal adenocarcinoma (PDAC). Compared with gemcitabine‐sensitive PDAC samples, gemcitabine‐resistant PDAC samples classified by 28‐GPS showed poorer overall survival, higher genomic instability, lower immune infiltration, higher metabolic level and higher‐fidelity DNA damage repair.

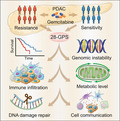

**Abbreviations**
28‐GPS: 28 gene pairs
AUC: area under curve
BH: Benjamini–Hochberg
CGP: cancer genome project
CNV: copy number variation
CR: complete response
CTRP: cancer therapeutics response portal
DDR: DNA damage response
DEGs: differentially expressed genes
DEMs: differentially expressed methylations
FDA: Food and Drug Administration
FDR: false discovery rate
GDSC: Genomics of Drug Sensitivity in Cancer
GEO: gene expression omnibus
HRDscore: homologous recombination defect score
ICGC: International Cancer Genome Consortium
KEGG: Kyoto Encyclopedia of Genes and Genomes
OS: overall survival
PACA‐AU: pancreatic cancer‐AU
PACA‐CA: pancreatic cancer‐CA
PD: progressive disease
PDAC: pancreatic ductal adenocarcinoma
PR: partial response
REO: relative expression ordering
ROC: receiver operating characteristic
SD: stable disease
ssGSEA: single sample gene set enrichment analysis
TCGA: The Cancer Genome Atlas
TCR: T‐cell receptors
TMB: tumour mutational burden

## 1 Introduction

Pancreatic ductal adenocarcinoma (PDAC), which accounts for the majority of pancreatic cancer, is a fatal disease with an extremely poor prognosis [1]. The 5‐year survival rate for PDAC is approximately 10%. Although radical surgical resection may increase the 5‐year survival rate to about 20%, most patients can not undergo surgery due to late‐stage diagnosis and metastases [2, 3]. Currently, gemcitabine‐based monotherapy or combination chemotherapy is still the standard treatment option for PDAC.

A phase III clinical trial showed that the clinical benefit rate of gemcitabine in the treatment of advanced pancreatic cancer reached 23.8%, and gemcitabine was widely used in clinics [4]. As a standard regimen for the treatment of pancreatic cancer, gemcitabine combined with drugs such as paclitaxel and erlotinib showed significantly improved survival in advanced pancreatic cancer [5, 6]. Among patients with resected or metastatic pancreatic cancer, the combination chemotherapy regimen consisting of oxaliplatin, irinotecan, fluorouracil and leucovorin (FOLFIRINOX) showed significantly longer survival than gemcitabine. However, it is worth noting that more drugs are accompanied by higher toxicities [7, 8]. Unfortunately, despite the use of adjuvant therapy, disease‐free survival or overall survival of PDAC has not been improved [9]. The poor prognosis of PDAC is mainly due to the majority of patients treated with gemcitabine chemotherapy eventually showing resistance [10].

Resistance to gemcitabine in PDAC is a complex biological process, and the underlying mechanism of resistance is not clear. It is well known that PDAC is a quite complex disease characterized by molecular and clinical heterogeneity. Focussing on a single PDAC driver gene such as *KRAS*, *TP53*, *SMAD4* or CDKN2A failed to predict whether patients are sensitive or resistant to gemcitabine [11, 12]. Genomic and epigenomic characteristics jointly regulate gene expression. Therefore, capturing the gene expression characteristics caused by genomic or epigenomic events may be more effective than analysing mutations in predicting the patient's response to gemcitabine. Up to date, the gemcitabine signatures derived from the transcriptome include 14‐gene signature [13] and GemPred signature [14]. However, current gemcitabine signatures consisting of a number of genes with different weights were developed based on absolute expression level of genes, which were limited by experimental batch effects, RNA degradation, sequencing platform differences, data normalization methods and so on [15]. Previous studies demonstrated that relative expression ordering (REO)‐based signatures were robust across different data sets [16]. Cheng *et al*. [17] have demonstrated that the REO patterns of gene pairs were insensitive to tumour purities of samples. Moreover, the REO‐based signature could be applied to the individual patient using within‐sample REOs [18]. Thus, our study aimed to develop the gene pair signature of gemcitabine response for PDAC based on REOs.

In this study, based on REO, we developed a qualitative transcriptional signature to predict the response to gemcitabine for PDAC. The prognostic performance of the signature was validated in multiple independent data sets. Finally, we conducted a systematic and comprehensive analysis to further explore the underlying mechanism of gemcitabine resistance.

## 2 Material and methods

### 2.1 PDAC data and preprocessing

The transcriptome data were downloaded from publicly available databases, including The Cancer Genome Atlas (TCGA), International Cancer Genome Consortium (ICGC, https://dcc.icgc.org/), ArrayExpress (https://www.ebi.ac.uk/arrayexpress/) and Gene Expression Omnibus (GEO, http://www.ncbi.nlm.nih.gov/geo/) (Table 1). PDAC samples from TCGA and ICGC were used as the training cohort to develop the gemcitabine signature. The validation data sets included GSE62452, GSE57495, GSE71729, GSE28735, GSE17891 and E‐MATB‐6134. The multiomics and drug information of TCGA were downloaded from cBioPortal (https://www.cbioportal.org/) and FIREHOSE (http://gdac.broadinstitute.org/). Each chemotherapy drug was accompanied with corresponding response information in the TCGA drug information. We only retained samples with response information to gemcitabine chemotherapy, and for samples with multiple rounds of gemcitabine treatments, we only kept the samples with first response information to gemcitabine. Pancreatic cancer‐CA (PACA‐CA) patients’ chemotherapy information was obtained from the ICGC. In our study, only PDAC samples with survival information were used. Silent mutations were excluded from TCGA mutation data. In PACA‐CA expression data, only tumour‐related specimens were retained. The probe‐level expression values of pancreatic cancer‐AU (PACA‐AU) and GEO data sets were annotated to gene‐level with matched platform information according to the following criteria: if multiple probe‐sets were mapped to the same gene, the gene expression values were averaged, and multiple gene expression values were mapped to the same probe‐set were excluded. In addition, the gene expression data were filtered by the HUGO Gene Nomenclature Committee database to retain the protein‐coding genes.

**Table 1:** The PDAC data sets used in this study.

Data	Data type	PDAC samples	Data source
PACA‐CA	mRNA	213	https://icgc.org/
PACA‐AU	mRNA	242	https://icgc.org/
GSE62452	mRNA	65	http://www.ncbi.nlm.nih.gov/geo
GSE57495	mRNA	63	http://www.ncbi.nlm.nih.gov/geo
GSE71729	mRNA	125	http://www.ncbi.nlm.nih.gov/geo
GSE28735	mRNA	45	http://www.ncbi.nlm.nih.gov/geo
GSE17891	mRNA	19	http://www.ncbi.nlm.nih.gov/geo
E‐MATB‐6134	mRNA	288	https://www.ebi.ac.uk/arrayexpress
TCGA	mRNA	146	https://www.cbioportal.org/
TCGA	Mutation	147	https://www.cbioportal.org/
TCGA	Methylation	152	https://www.cbioportal.org/
TCGA	DNA copy number	152	https://www.cbioportal.org/

### 2.2 Cell line data and preprocessing

Gene expression data of PDAC cell lines were downloaded from the Cancer Dependency Map (https://depmap.org/portal/) and corresponding annotation files were obtained from the Cancer Cell Line Encyclopedia (CCLE, https://sites.broadinstitute.org/ccle) (Table S1). Only primary ductal adenocarcinoma cell lines were retained. Gene expression data were directly used after retaining 49 PDAC cell lines. In addition, pharmacological data of gemcitabine were downloaded from the Genomics of Drug Sensitivity in Cancer (GDSC, https://www.cancerrxgene.org), the Cancer Therapeutics Response Portal (CTRP, https://ctd2‐data.nci.nih.gov/Public/Broad) and the work of Cancer Genome Project (CGP) [19] (Table S2).

### 2.3 Single‐cell data and preprocessing

Single‐cell data of 24 PDAC tumour samples were obtained from Peng *et al*. [20]. r package ‘Seurat’ (v4.0.4) was used for data preprocessing and subsequent analysis [21]. All functions were run with default parameters. To filter low‐quality cells, only cells with ≥ 1000 transcripts per cell, ≥ 3 cells per transcript and ≤ 10% mitochondrial transcripts were included for the following analysis. Cell type identification was performed using known cell type markers derived from the literature or the CellMarker and PanglaoDB databases [20, 22, 23].

### 2.4 Development of the REO‐based gemcitabine signature

In the TCGA data set, PDAC samples were classified into the resistant and sensitive groups based on their response to gemcitabine, where patients with complete response (CR), partial response (PR) and stable disease (SD) comprised the sensitive group and progressive disease (PD) belonged to the resistant group. Differentially expressed genes (DEGs) were identified between gemcitabine‐resistant and ‐sensitive groups using the Wilcoxon rank‐sum test. For a gene pair composed of DEGs, G_*i*_ and G_*j*_ were used to represent the expression value of gene *i* and gene *j*, respectively. Fisher's exact test was used to evaluate whether the frequency of a specific REO pattern (G_*i*_ > G_*j*_ or G_*i*_ < G_*j*_) in the resistant group was significantly higher than the frequency in the sensitive group (*P* < 0.05). A panel of DEG‐related gene pairs was screened out by pairwise comparisons of all DEGs. To improve the accuracy of the prediction, DEG‐related gene pairs were filtered by maintaining the consistency of the REO pattern and the direction of average rank difference. Using univariate Cox proportional hazards model, the DEG‐related gene pairs were further filtered in TCGA, PACA‐CA and PACA‐AU data sets to obtain a set of prognosis‐related gene pairs. Finally, the common gene pairs of the three data sets were defined as the gemcitabine signature.

### 2.5 Defining resistant and sensitive sample

The classification threshold was obtained from the receiver operating characteristic (ROC) curve, which was drawn by the r package ‘pROC’. A single sample was classified into the resistant group when the number of 28‐GPS voting for resistance was no less than the classification threshold; otherwise, the sensitive group. Specifically, for a given gene pair in 28‐GPS, if G_*i*_ was greater than G_*j*_, it would be scored as 1. Then, the scores of all gene pairs in 28‐GPS were added and recorded as resistance score. The classification threshold should be correspondingly adjusted with the number of matched gene pairs within the sample.

### 2.6 Multiomics feature analysis

Fisher's exact test was used to identify genes with significantly differential mutation frequencies between gemcitabine‐resistant and ‐sensitive groups. The Wilcoxon rank‐sum test was applied to detect DEGs and differentially expressed methylations (DEMs) between the two groups with a false discovery rate (FDR) < 0.05. The *P* value was adjusted using the Benjamini–Hochberg (BH) procedure. Tumour mutational burden (TMB) was calculated based on the number of nonsynonymous somatic mutations. Homologous recombination defect score (HRDscore) was derived from the work of Thorsson *et al*. [24]. TMB, HRDscore and copy number variation (CNV) between the two groups were compared using the Wilcoxon rank‐sum test.

### 2.7 Functional enrichment analysis

Functional enrichment analysis of the genes with corresponding deregulation direction of DEGs and DEMs was performed using the r package ‘clusterProfiler’ (version 4.0.5) [25]. Metabolism and immune system‐related pathways were obtained from the Kyoto Encyclopedia of Genes and Genomes (KEGG) database. A panel of DNA damage response (DDR) related genes was collected from the KEGG database and two published literatures [26, 27]. Subsequently, the enrichment scores of the metabolic pathway, immune pathway and DDR pathway for each sample were calculated using single sample gene set enrichment analysis (ssGSEA).

### 2.8 Immune infiltration analysis

The proportion of infiltrating immune cells in the tumour immune microenvironment was estimated using transcriptome‐based algorithms CIBERSORTx [28], EPIC [29], TIMER [30], QUANTISEQ [31] and XCELL [32] on the Website CIBERSORTx (https://cibersortx.stanford.edu/) and TIMER 2.0 (http://timer.cistrome.org/). T‐cell receptors (TCR) richness was obtained from Thorsson *et al*. [24]. Immune‐related signatures were obtained from Chen *et al*. [33]. The Wilcoxon rank‐sum test was used to compare the difference in immune cell infiltration proportion, TCR richness and immune‐related signature scores between gemcitabine‐resistant and ‐sensitive groups. Forty‐five immune checkpoint genes with known activation or inhibition effects were obtained from Auslander *et al*. [34]. Differentially expressed immune checkpoint genes were identified using the Wilcoxon rank‐sum test.

### 2.9 Network analysis

Spearman rank correlation, with ¦*r*¦ > 0.4 and *P* < 0.05, was used to calculate the correlation between DEGs and differentially expressed immune checkpoint genes or differentially expressed DDR genes. The correlations among metabolic pathway, immune pathway and DDR type were also calculated by Spearman rank correlation. cytoscape software (version 3.8.2, https://cytoscape.org/) was used to visualize the correlation network.

### 2.10 Survival analysis

The overall survival time curves were estimated by the Kaplan–Meier method, and tested using the log‐rank test.

### 2.11 Single‐cell CNV analysis

CNV analysis was carried out using the r Package ‘InferCNV’ [35]. The CNVs were calculated for each sample by expression level from single‐cell sequencing data with following parameters: cut‐off = 0.1, cluster_by_groups = TRUE, denoise = TRUE and HMM = TRUE. The cells, except ductal and acinar cells, were used as reference cells. For each sample, the CNV score of each cell was calculated as a quadratic sum of CNVregion ‐ 1.

### 2.12 Cell communication analysis

Cell–cell communication was investigated via the r package ‘CellChat’ (v1.1.3) [36]. After creating CellChat objects, we set the Secreted Signalling pathways as the reference database and used default parameters to identify putative interaction pairs.

### 2.13 Statistical analysis

All statistical analyses in this study were performed using r software (v 4.1.1). The significance of the *P* value is shown in the following way: *P < 0.05; **P < 0.01; ***P < 0.001; ns: No significance.

## 3 Results

### 3.1 REO‐based gemcitabine signature for PDAC

The steps of identifying the gemcitabine signature are summarized in the flow chart (Fig. S1). Based on the response to gemcitabine, PDAC samples were divided into resistant and sensitive groups, and 282 DEGs were identified between the two groups (*P* < 0.05, Wilcoxon rank‐sum test). Among all gene pairs composed of DEGs, we screened out 2516 candidate gene pairs (*P* < 0.05, Fisher's exact test), of which 1028 gene pairs had REO patterns consistent with the direction of average rank difference. Subsequently, 407, 153 and 164 prognosis‐related gene pairs were identified from the TCGA, PACA‐CA and PACA‐AU data sets, respectively (*P* < 0.05, univariate Cox regression model). Finally, the common 28 gene pairs (28‐GPS) of the three data sets were defined as gemcitabine signature (Table S3).

### 3.2 Gemcitabine‐resistant samples classified by 28‐GPS showed worse prognosis

**Fig. 1:**
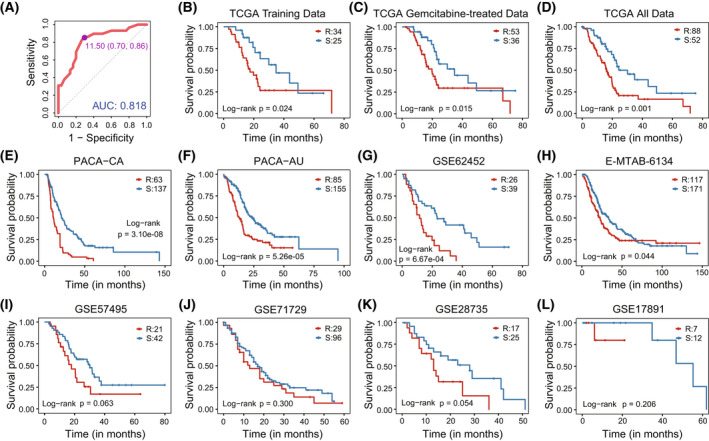
The prognostic performance of 28‐GPS. 28‐GPS: 28 gene pairs. (A) The ROCs for the TCGA training cohort (59 samples). The purple point represents the best classification threshold. ROC, receiver operating characteristic; TCGA, the cancer genome atlas; (B–D) Kaplan–Meier survival curves show the OS difference between gemcitabine‐resistant and ‐sensitive PDAC samples classified by 28‐GPS in TCGA training data (R: 34; S: 25), TCGA gemcitabine‐treated data (R: 53; S: 36) and TCGA all data (R: 88; S: 52). OS: Overall survival; PDAC, pancreatic ductal adenocarcinoma; R, resistant samples; S, sensitive samples. (E–L) Kaplan–Meier survival curves show the OS difference between gemcitabine‐resistant and ‐sensitive PDAC samples classified by 28‐GPS in PACA‐CA (R: 63; S: 137), PACA‐AU (R: 85; S: 155), GSE62452 (R: 26; S: 39), E‐MTAB‐6134 (R: 117; S: 171), GSE57495 (R: 21; S: 42), GSE71729 (R: 29; S: 96), GSE28735 (R: 17; S: 25) and GSE17891 (R: 7; S: 12). PACA‐AU, pancreatic cancer‐AU; PACA‐CA, pancreatic cancer‐CA; R, resistant samples; S, sensitive samples. [Colour figure can be viewed at wileyonlinelibrary.com]

According to the classification threshold 11.5 derived from the ROC in TCGA training set (Fig. 1A), resistant samples classified by 28‐GPS showed significantly poorer overall survival (OS) than sensitive samples (*P* = 0.024, log‐rank test, Fig. 1B). Furthermore, in TCGA gemcitabine‐treated and all samples, resistant samples were also accompanied with a worse prognosis (*P* = 0.015 for gemcitabine‐treated samples and *P* = 0.001 for all samples, log‐rank test, Fig. 1C,D). Moreover, in independent PDAC samples from the GEO, ArrayExpress and ICGC, the Kaplan–Meier survival curve uncovered poorer OS in resistant samples classified by 28‐GPS (PACA‐CA: *P* = 3.10e‐08; PACA‐AU: *P* = 5.26e‐05; GSE62452: *P* = 6.67e‐04; E‐MTAB‐6134: *P* = 0.044; GSE57495: *P* = 0.063; GSE71729: *P* = 0.300; GSE28735: *P* = 0.054; GSE17891: *P* = 0.206; log‐rank test, Fig. 1E–L).

### 3.3 28‐GPS could predict the response to gemcitabine in PDAC cell lines

**Fig. 2:**
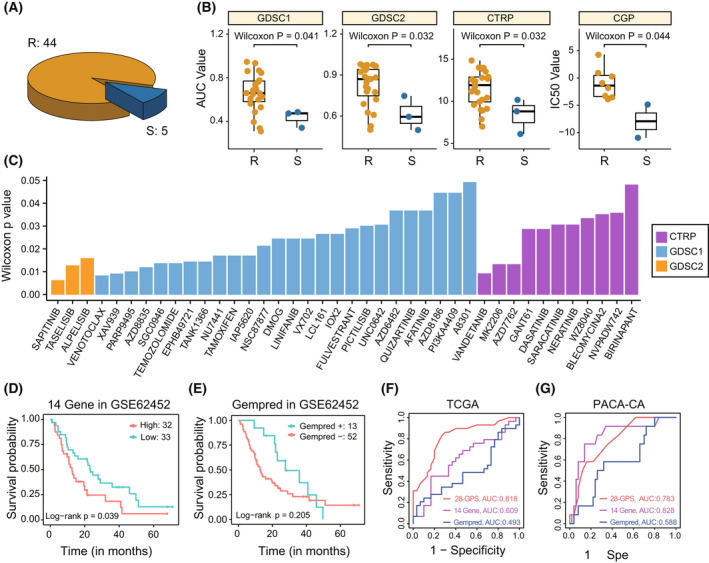
Performance of 28‐GPS in cell lines and comparison of 28‐GPS with other signatures. 28‐GPS, 28 gene pairs; (A) The number of cell lines classified into the resistant group or the sensitive group based on 28‐GPS (R: 44; S: 5). R, resistant cell lines; S, sensitive cell lines. (B) Boxplot shows the difference in the response to gemcitabine between gemcitabine‐resistant and ‐sensitive PDAC cell lines in GDSC, CTRP and CGP. The Wilcoxon rank‐sum test was used to calculate the statistical significance of GDSC1 (R: 23; S: 3), GDSC2 (R: 23; S: 3), CTRP (R: 25; S: 3) and CGP (R: 8; S: 2). In the boxplot, the centre line represents the median and the upper and lower lines represent the upper and lower quartiles. The vertical line reaches the maximum and minimum values. Each dot represents a cell line. CGP, cancer genome project; CTRP, cancer therapeutics response portal; GDSC, genomics of drug sensitivity in cancer; PDAC, pancreatic ductal adenocarcinoma; R, resistant cell lines; S, sensitive cell lines. (C) Barplot shows the drugs with pharmacological value difference between gemcitabine‐resistant and ‐sensitive PDAC cell lines in CTRP (purple; R: 25; S: 3), GDSC1 (blue; R: 23; S: 3) and GDSC2 (yellow; R: 23; S: 3). Rows represent *P* values and columns represent drugs. (D, E) Kaplan–Meier survival curves show the OS difference between the two groups classified by 14‐gene signature (D; high: 32; low: 33) and Gempred signature (E; Gempred +: 13; Gempred −: 52) in GSE62452. OS, overall survival; (F, G) Comparison of the classification performance of 28‐GPS (red), 14‐gene signature (purple), and Gempred signature (blue) in the TCGA (F; samples: 59) and PACA‐CA (G; samples: 62). PACA‐CA, pancreatic cancer‐CA. [Colour figure can be viewed at wileyonlinelibrary.com]

Among the 49 PDAC cell lines from the CCLE database, 44 cell lines were classified into the resistant group and five cell lines were classified into the sensitive group based on 28‐GPS (Fig. 2A). For PDAC cell lines treated with gemcitabine in GDSC1, resistant cell lines classified by 28‐GPS presented higher AUC values than sensitive cell lines (*P* = 0.041, Wilcoxon rank‐sum test, Fig. 2B). Similar results were found in GDSC2 and CTRP data sets, respectively (GDSC2: *P* = 0.032; CTRP: *P* = 0.032; Wilcoxon rank‐sum test, Fig. 2B). In CGP data, resistant cell lines classified by 28‐GPS presented higher IC50 values than sensitive cell lines (*P* = 0.044, Wilcoxon rank‐sum test, Fig. 2B).

In the GDSC and CTRP data sets, a group of drugs whose pharmacological values in resistant cell lines were significantly lower than that in sensitive cell lines was obtained (*P* < 0.05, Wilcoxon rank‐sum test, Fig. 2C). Some of these drugs are phosphoinositide 3‐kinase (PI3K) inhibitors, such as taselisib, alpelisib, pictilisib, AZD6482 and AZD8186 (Fig. 2C). The PI3K inhibitor taselisib showed higher potency against PIK3CA‐mutant tumours and inhibition of the PI3K pathway could be a target for PDAC [37, 38]. In addition, the novel Akt inhibitor MK2206 combined with gemcitabine demonstrated inhibitory effect on Akt phosphorylation at the cell line level [39]. The DNA‐PK inhibitor NU7441and Chk1 inhibitor AZD7762 were proved to be potential combinational partners of gemcitabine [40, 41].

### 3.4 28‐GPS showed better performance than other gemcitabine signatures

Two published gemcitabine signatures, 14‐Gene signature and Gempred signature, have been reported [13, 14]. We compared the survival differences between samples classified by 28‐GPS, 14‐Gene signature and Gempred signature in GSE62452, and the results showed that 28‐GPS had the best prognostic classification performance (28‐GPS: *P* = 6.67e‐04, Fig. 1G; 14‐Gene signature: *P* = 0.039; Gempred signature: *P* = 0.205, log‐rank test, Fig. 2D‐2E). Then, the area under curve (AUC) value of the ROC was used to assess the binary classification performance of these three signatures. The highest classification accuracy was achieved by the 28‐GPS (AUC = 0.818) in the TCGA data set (Fig. 2F). In addition, the 28‐GPS reached the second highest classification accuracy (AUC = 0.783) in the PACA‐CA data set (Fig. 2G). Although no genes were overlapped among 28‐GPS, 14‐gene signature and Gempred signature, all the signatures were related to the lysosome pathway, where inhibition of lysosome could enhance gemcitabine‐induced apoptosis [42] (Fig. S2A,B).

### 3.5 Gemcitabine‐resistant samples classified by 28‐GPS showed high genomic instability

**Fig. 3:**
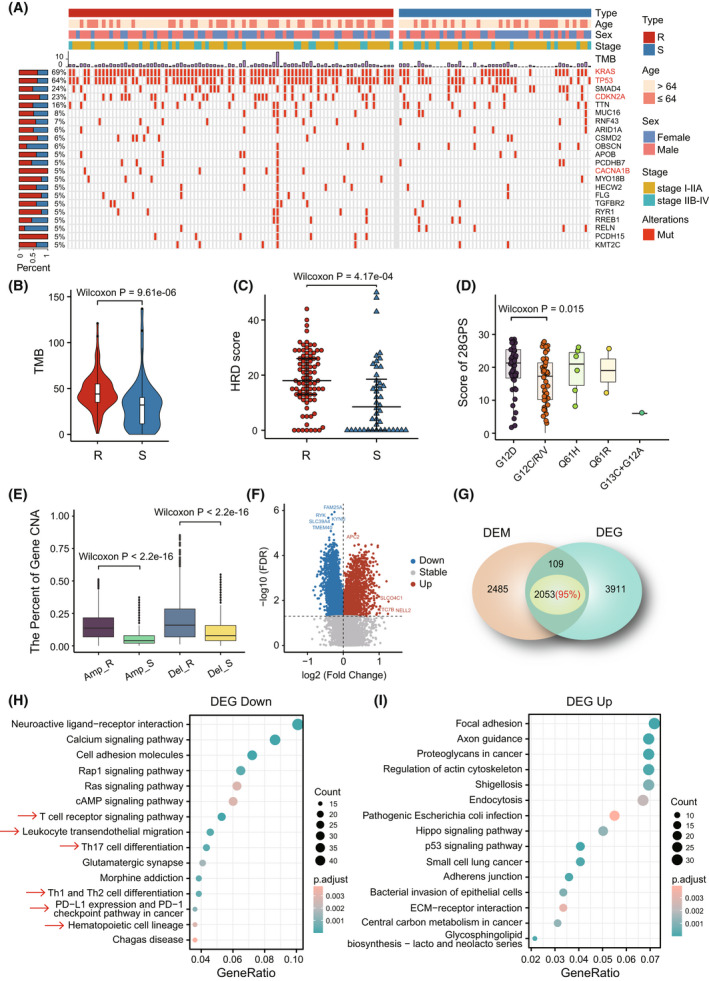
The multiomics landscape between gemcitabine‐resistant and ‐sensitive PDAC samples classified by 28‐GPS in TCGA. PDAC, pancreatic ductal adenocarcinoma; 28‐GPS, 28 gene pairs; TCGA, the cancer genome atlas; (A) Oncoplot shows 22 genes with mutation frequencies not less than 5% in the TCGA (R: 88; S: 52). Rows represent genes and columns represent samples. The genes in the red font indicate that the mutation frequencies in the resistant samples are significantly higher than that in the sensitive samples (*P* < 0.05, Fisher's exact test). The left bar shows the mutation percentage between gemcitabine‐resistant and ‐sensitive samples and the top bar shows the total number of mutations. Clinical characteristics are also provided at the top. R, resistant samples; S, sensitive samples. (B) TMB difference between gemcitabine resistant and sensitive samples in the TCGA (R: 88; S: 52). In the violin plot, the centre line represents the median and the upper and lower lines represent the upper and lower quartiles. The vertical line reaches the maximum and minimum values. TMB, tumour mutational burden; R, resistant samples; S, sensitive samples. (C) HRDscore difference between gemcitabine resistant and sensitive samples in the TCGA (R: 88; S: 52). The centre line represents the median and the upper and lower lines represent the upper and lower quartiles. Each dot represents a sample. HRDscore, homologous recombination defect score; R, resistant samples; S, sensitive samples. (D) 28‐GPS resistance scores of different KRAS somatic mutations in TCGA (G12D: 43; G12C/R/V: 45; Q61H: 6; Q61R: 2; G13C + G12A: 1). The centre line represents the median and the upper and lower lines represent the upper and lower quartiles. The vertical line reaches the maximum and minimum values. Each dot represents a sample. (E) The amplification and deletion difference of CNV between gemcitabine resistant and sensitive samples in the TCGA (R: 88; S: 52). The centre line represents the median and the upper and lower lines represent the upper and lower quartiles. The vertical line reaches the maximum and minimum values. CNV, copy number variation; Amp_R, amplification in resistant samples; Amp_s, amplification in sensitive samples; Del_R, deletion in resistant samples; Del_S, deletion in sensitive samples; (F) Volcano plot depicts hypermethylated and hypomethylated genes between gemcitabine resistant and sensitive samples in the TCGA (R: 88; S: 52). The horizontal dotted line shows the adjusted *P* value of 0.05. The vertical dotted line shows the fold change value of 1. (G) Venn map shows the intersection of DEMs and DEGs. DEMs, differentially expressed methylations; DEGs, differentially expressed genes; (H, I) KEGG pathway enrichment analyses on the intersection genes of DEMs and DEGs, representing underexpressed and hypermethylated genes (H), and overexpressed and hypomethylated genes (I), respectively. The red arrows represent immune‐related pathways. DEGs, differentially expressed genes; DEMs, differentially expressed methylations. [Colour figure can be viewed at wileyonlinelibrary.com]

In the TCGA data set, among the genes whose mutation frequencies were not less than 5%, resistant samples classified by 28‐GPS showed significantly higher mutation frequencies in three PDAC driver genes: *KRAS*, *TP53* and *CDKN2A* (*P* < 0.05, Fisher's exact test, Fig. 3A). The resistant samples also displayed significantly higher TMB (*P* = 9.61e‐06, Wilcoxon rank‐sum test, Fig. 3B) and HRDscore (*P* = 4.17e‐04, Wilcoxon rank‐sum test, Fig. 3C) than the sensitive samples. The resistance score of 28‐GPS was applied to investigate resistant mechanisms underlying mutation of four driver genes in PDAC (*KRAS*, *TP53*, *SMAD4* and *CDKN2A*). As a frequent *KRAS* mutation type observed in PDAC, we found that p.G12D mutation exhibited significantly higher resistance scores of 28‐GPS than other types of mutation (*P* = 0.015, Wilcoxon rank‐sum test, Fig. 3D). In addition, samples with p.P278S and p.Q38* in *TP53* and p.Y44* in *CDKN2A* reached the highest scores of 28‐GPS respectively, which was not observed in *SMAD4* (Fig. S2C–E). CNV analysis showed the frequencies of amplification and deletion in the resistant samples were significantly higher than those in the sensitive samples (*P* < 2.20e‐16, Wilcoxon rank‐sum test, Fig. 3E, Fig. S2F).

By comparing the methylation profiles between gemcitabine‐resistant and ‐sensitive PDAC samples classified by 28‐GPS in TCGA, 1720 hypermethylated and 2613 hypomethylated genes were detected, respectively (FDR < 0.05, Wilcoxon rank‐sum test, Fig. 3F). Subsequently, 2053 genes with corresponding deregulation direction with DEGs were retained (Fig. 3G, Fig. S2G,H). KEGG pathway enrichment analysis showed that 1035 underexpressed and hypermethylated genes were enriched in multiple immune‐related pathways, such as T‐cell receptor signalling pathway, and leukocyte transendothelial migration (Fig. 3H). In addition, the 1018 overexpressed and hypomethylated genes were not only enriched in the immune‐related pathway, but also enriched in metabolic pathways, such as central carbon metabolism and glycosphingolipid biosynthesis (Fig. 3I).

### 3.6 Gemcitabine‐resistant samples classified by 28‐GPS showed low immune infiltration

**Fig. 4:**
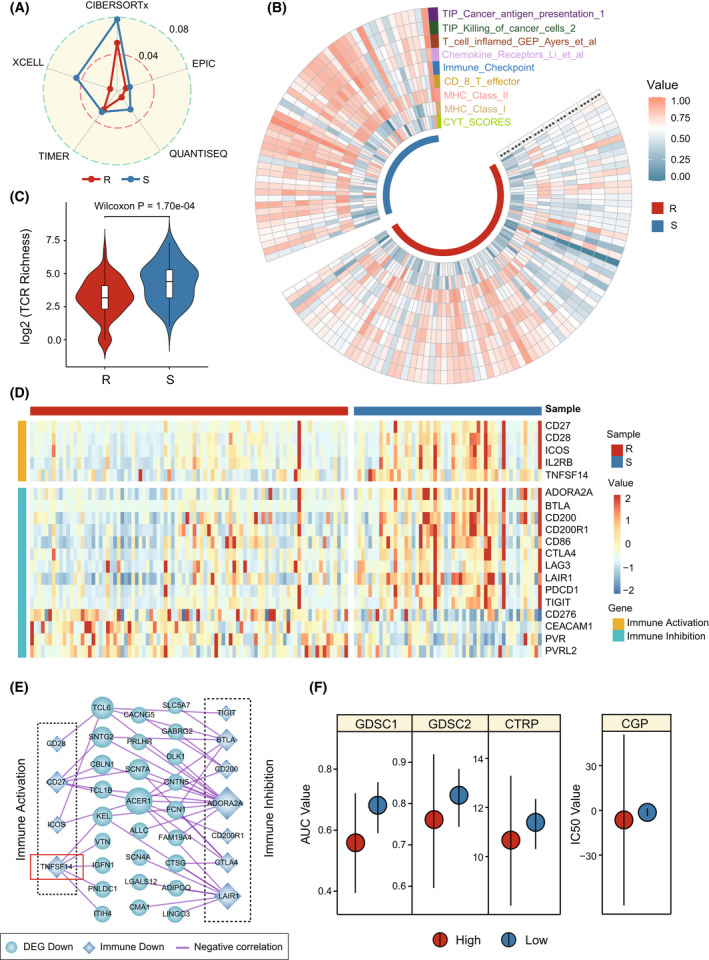
The immune landscape between gemcitabine‐resistant and ‐sensitive samples classified by 28‐GPS in TCGA. 28‐GPS, 28 gene pairs; TCGA, The cancer genome atlas; (A) Radar chart shows the proportion of CD8^+^ T cells identified by five immune infiltration assessment algorithms between gemcitabine‐resistant and ‐sensitive samples. R, resistant samples; S, sensitive samples. (B) Comparison of immune‐related signatures between gemcitabine resistant and sensitive samples in the TCGA (R: 87; S: 51; ***P < 0.001, Wilcoxon rank‐sum test). R, resistant samples; S, sensitive samples. (C) TCR richness difference between gemcitabine‐resistant and ‐sensitive samples in the TCGA (R: 88; S: 52). The centre line represents the median and the upper and lower lines represent the upper and lower quartiles. The vertical line reaches the maximum and minimum values. TCR, T cell receptors; R, resistant samples; S, sensitive samples. (D) Heatmap displays 19 differentially expressed immune checkpoint genes in the TCGA (R: 88; S: 52). R, resistant samples; S, sensitive samples. (E) The correlation network between differentially expressed immune checkpoint genes and DEGs. DEGs, differentially expressed genes; (F) Dot plot shows the difference in the response to gemcitabine between *TNFSF14* high and low expression PDAC cell lines in GDSC1 (high: 8; low: 18), GDSC2 (high: 8; low: 18), CTRP (high: 6; low: 22) and CGP (high: 2; low: 8). The point represents the median. The vertical line reaches the maximum and minimum values. CGP, cancer genome project; CTRP, cancer therapeutics response portal; GDSC, genomics of drug sensitivity in cancer; PDAC, pancreatic ductal adenocarcinoma. [Colour figure can be viewed at wileyonlinelibrary.com]

**Fig. 5:**
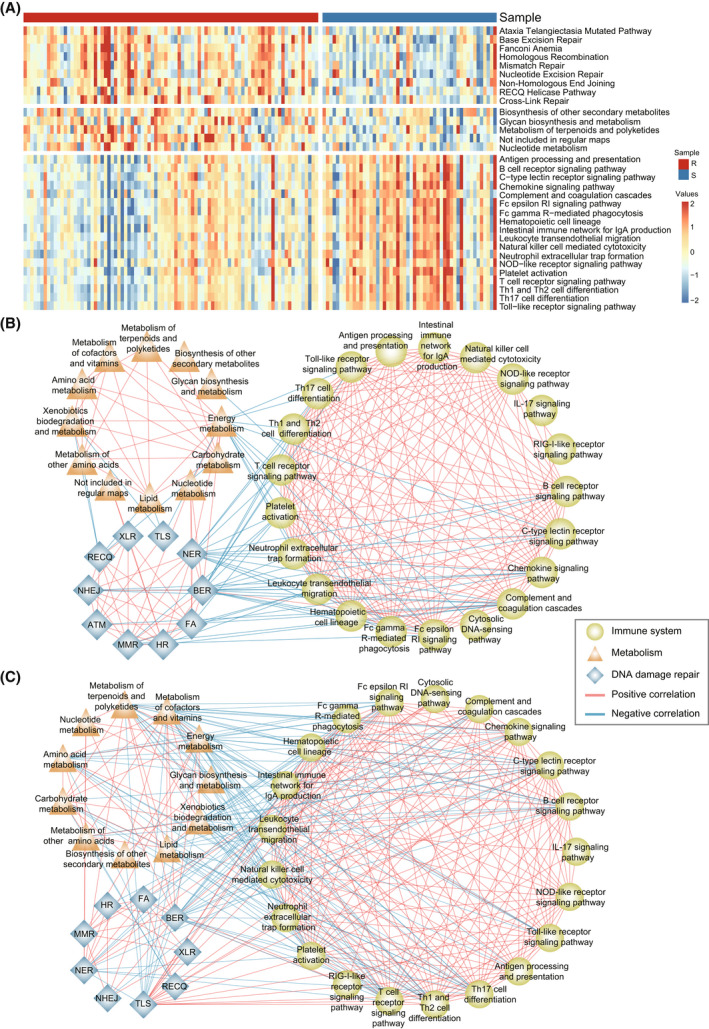
The DDR, metabolic and immune‐related pathway landscape between gemcitabine‐resistant and ‐sensitive samples classified by 28‐GPS in TCGA. DDR, DNA damage response; 28‐GPS, 28 gene pairs; TCGA, the cancer genome atlas; (A) Heatmap shows the ssGSEA enrichment scores of DDR, metabolism and immunity pathways. ssGSEA, single sample gene set enrichment analysis; R, resistant samples; S, sensitive samples. (B, C) The correlation network between DDR, metabolic and immune‐related pathways in resistant group (B) and sensitive group (C). [Colour figure can be viewed at wileyonlinelibrary.com]

The above KEGG pathway enrichment results inspired us to investigate the difference in immunity between gemcitabine‐resistant and ‐sensitive PDAC samples. Here, five current transcriptome‐based assessment algorithms for immune cell infiltration were applied to estimate the fraction of infiltrating immune cells in TCGA samples (Fig. 4A). Compared with sensitive samples, resistant samples classified by 28‐GPS showed a consistent low immune infiltration of CD8^+^ T cells in the five algorithms (Fig. 4A, Fig. S3). The CD4^+^ T cells exhibited low immune infiltration by TIMER and EPIC in the gemcitabine‐resistant samples (Fig. S3). By comparing the immune‐related signatures, resistant samples displayed significantly lower immune‐related signature scores (*P* < 0.05, Wilcoxon rank‐sum test, Fig. 4B). We also observed significantly lower TCR richness levels (*P* = 1.70e‐4, Wilcoxon rank‐sum test, Fig. 4C) and immune system scores (*P* < 0.05, Wilcoxon rank‐sum test, Fig. 5a) in the resistant PDAC samples.

Among 45 immune checkpoint genes, 19 were detected to be differentially expressed and generally downregulated in the resistant samples (*P* < 0.05, Wilcoxon rank‐sum test, Fig. 4D). The correlation network between differentially expressed immune checkpoint genes and DEGs was constructed to further explore potential mechanisms involved in resistance to gemcitabine. We identified a differential correlation network in the resistant group and only retained the DEGs with a log2 fold change > 1.5 (Fig. 4E). Among the immune genes with activation effects, we found DEGs frequently interacted with *TNFSF14*, which was primarily expressed on activated T cells, activated natural killer (NK) cells, and immature dendritic cells (DC). In addition, PDAC cell lines with high *TNFSF14* expression were accompanied by high AUC or IC50 values of gemcitabine (Fig. 4F).

### 3.7 Gemcitabine‐resistant samples classified by 28‐GPS showed high‐fidelity DNA damage repair

As gemcitabine mainly interfered with DNA synthesis and the metabolism also affected drug response, we tried to explore the resistant mechanism of gemcitabine from the perspective of DNA damage repair (DDR) and metabolism. Using ssGSEA, resistant samples classified by 28‐GPS reached significantly higher DDR and metabolic pathway enrichment scores than sensitive samples (*P* < 0.05, Wilcoxon rank‐sum test, Fig. 5A). Combined with the immune difference observed above, the correlation analysis between DDR, metabolic and immune‐related pathways was conducted on the resistant group and sensitive group, respectively. The sensitive group showed frequent associations between different pathways. As a tolerant DNA damage repair process, translesion synthesis (TLS) had multiple positive correlations with immune‐related pathways in the sensitive group, which did not appear in the resistant group (Fig. 5B,C).

### 3.8 Single‐cell analysis revealed the intra‐ and inter‐tumoural heterogeneity

**Fig. 6:**
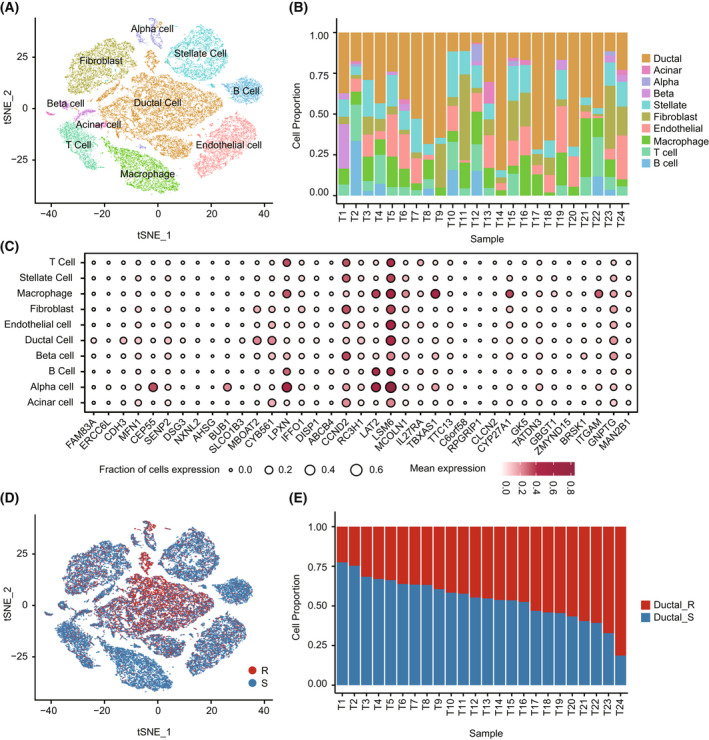
The single‐cell heterogeneity of 24 PDAC samples. PDAC, pancreatic ductal adenocarcinoma; (A) TSNE visualization of 42 063 cells analysed by scRNA‐seq and integrated across 24 PDAC samples. Clusters were annotated for cell types using canonical markers. (B) Barplot shows the relative proportions of cell types across 24 PDAC samples. (C) Bubble plot shows the expression pattern of genes in 28‐GPS in each cell type, with cell types in rows and genes in columns. The size of each bubble represents the fraction of cells with expressed corresponding genes and colour represents the level of gene expression. 28‐GPS, 28 gene pairs; (D) TSNE visualization of 42 063 cells classified by 28‐GPS. R, resistant subtype; S, sensitive subtype. (E) Barplot shows the relative proportion of resistant and sensitive ductal cells across 24 PDAC samples. Ductal_R, resistant ductal cell; Ductal_S, sensitive ductal cell. [Colour figure can be viewed at wileyonlinelibrary.com]

In light of the immune difference between gemcitabine‐resistant and ‐sensitive PDAC samples, we aimed to investigate the effect of cell composition heterogeneity on gemcitabine resistance at the single‐cell level. After quality control, 42 063 cells from PDAC samples were retained and annotated into 10 clusters (Fig. 6A). Canonical cell markers were used to identify the cell types of different clusters (Fig. S4A). We found that cell composition presented substantial heterogeneity among different samples (Fig. 6B). Through CNV analysis, we observed elevated CNV scores in the ductal populations (Fig. S4B). Although the majority of genes in 28‐GPS had similar expression patterns across all cell types, there were also some cells that specifically expressed genes, such as *CEP55* for Alpha cell (Fig. 6C). Using 28‐GPS, ductal cells were classified into two subtypes (Fig. 6D), and the ductal cells in the same sample showed both the characteristics of sensitivity and resistance to gemcitabine (Fig. 6E), which was consistent with a recent report from Lee *et al*. [43].

### 3.9 Cell–cell communication in the resistant group could promote progression of PDAC

**Fig. 7:**
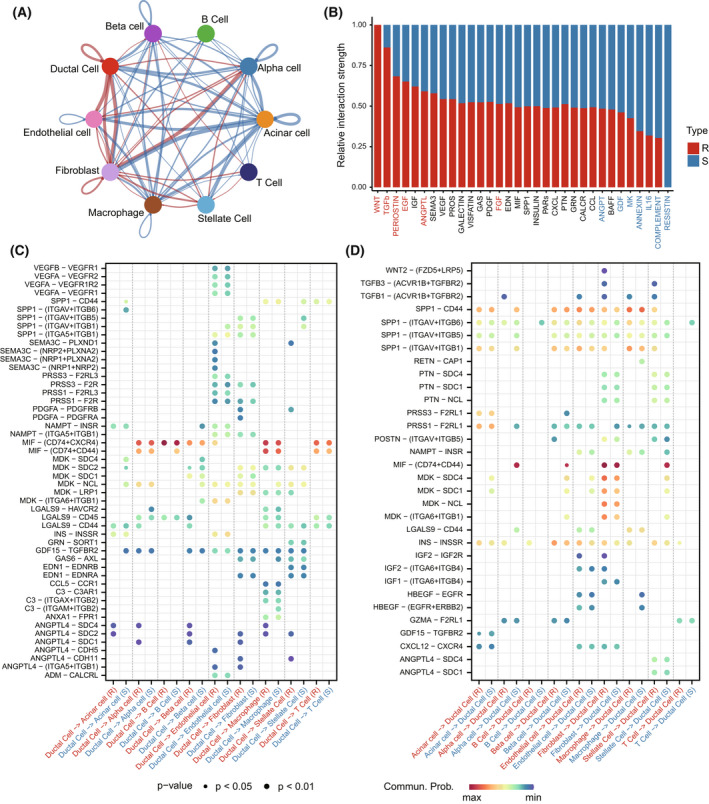
The cell–cell communication between gemcitabine‐resistant and ‐sensitive groups. (A) Overview of the comparison of ligand–receptor interactions between gemcitabine‐resistant and ‐sensitive groups. Red represents the resistant group had more interactions than the sensitive group, otherwise blue. The line thickness was proportional to the number of interaction difference. (B) Barplot shows the relative proportion of interaction strength for each signalling pathway between gemcitabine‐resistant and ‐sensitive groups (R:10440; S: 31623). The top signalling pathways coloured red are enriched in the resistant group, and signalling pathways coloured blue are enriched in the sensitive group. R, resistant subtype; S, sensitive subtype. (C, D) Comparison of the ligand‐receptor pairs in the communication of ductal cells to stromal cells (C) and the communication of stromal cells to ductal cells (D) between gemcitabine‐resistant and ‐sensitive groups. *P* values are indicated by circle size and communication probabilities are indicated by circle colour. [Colour figure can be viewed at wileyonlinelibrary.com]

In addition to the intrinsic cell information, cell–cell communication might also have effect on gemcitabine resistance. We found that there was frequent communication between ductal cells and fibroblasts in the resistant group, while in the sensitive group, ductal cells had frequent communication with macrophages (Fig. 7A). However, there was no difference in ductal cells communication with T cells between gemcitabine‐resistant and ‐sensitive groups (Fig. 7A). Next, the context‐specific signalling pathways were identified between gemcitabine‐resistant and ‐sensitive groups by comparing the interaction strength for each signalling pathway. Signalling pathways such as WNT and TGFβ were specifically active in the resistant group (Fig. 7B). Specific to TGFβ signalling pathway, ligand *TGFB3* and *TGFB1* with their multi‐subunit receptor *ACVR1B*/*TGFBR2* were active in resistant group from fibroblasts and stellate cells to ductal cells. In contrast, ligand *ANGPTL4* and multiple receptors, such as *SDC2*, *SDC1* and *CDH11*, were active in the resistant group from ductal cells to fibroblasts (Fig. 7C‐7D). Ligand *SPP1* and its multi‐subunit receptor *ITGAV*/*ITGB6* were found to be highly active in the sensitive group from T cells to ductal cells (Fig. 7D). In addition, ligand–receptor pair *HBEGF*‐*EGFR* was also found to act as major signalling from macrophages to ductal cells (Fig. 7D).

## 4 Discussion

Identifying the response signature of PDAC to gemcitabine is essential in determining the chemotherapy regimen. Here, we developed a qualitative gemcitabine signature for PDAC based on transcriptome, termed as 28‐GPS. Compared with existing signatures, 28‐GPS is robust to sequencing platforms or batch effects, and can be applied to PDAC at the individual level. Compared with gemcitabine‐sensitive PDAC samples, gemcitabine‐resistant PDAC samples classified by 28‐GPS showed lower immune infiltration, such as CD8^+^ T cell. Single‐cell analysis indicated cancer cells in the same PDAC sample showed both the characteristics of sensitivity and the resistance to gemcitabine.

In this study, the results suggest some possible combination regimens for PDAC. In the TCGA data, resistant samples classified by 28‐GPS showed lower immune infiltration and down‐regulation of the immune checkpoint genes. PDAC cell lines with higher expression of immune activating gene *TNFSF14* were sensitive to gemcitabine, suggesting that activating immunity might benefit PDAC from the treatment with gemcitabine. Chimeric antigen receptor T cells (CAR‐T) likewise serve as a hot spot for immunotherapy and two CAR‐T drugs Kymriah and Yescarta have been approved by the Food and Drug Administration (FDA) [44, 45]. An ongoing phase II trial indicated that CD8^+^ cells with targeting *KRAS* mutation showed effective treatment against cancer with mutant *KRAS* G12D [46]. And, PDAC samples with *KRAS* G12D in our study achieved a higher 28‐GPS resistance score. Therefore, gemcitabine combined with CAR‐T may be a promising approach for PDAC treatment. Genomic analysis in TCGA indicated that resistant samples classified by 28‐GPS displayed higher genomic instability, such as high HRDscore. In addition, as a tolerant DNA damage repair process accompanied by mutagenesis [47], TLS was found to be frequently and positively related to immune‐related pathways in sensitive samples, suggesting that lacking high‐fidelity DNA damage repair mechanisms might have a combined effect with gemcitabine to treat PDAC. And, the POLO (Pancreatic Cancer Olaparib Ongoing) trial has demonstrated that the olaparib group had significantly longer survival compared to the placebo group (7.4 months vs. 3.8 months) [48].

Although some immune cells showed inconsistent infiltration proportion between gemcitabine‐resistant and ‐sensitive samples, most of the immune cell infiltration presented no statistical significance. For example, Tregs exhibited high immune infiltration by CIBERSORTx and low immune infiltration by XCELL and QUANTISEQ in gemcitabine‐resistant samples, but the differences in Tregs infiltration showed no significance between resistant and sensitive samples detected by CIBERSORTx and XCELL (Fig. S3). A comprehensive evaluation of different immune infiltration methods by Sturm *et al*. [49] showed that there were differences in method performance between different cell types. Therefore, the consistent results produced by five immune infiltration methods were considered with high confidence in our work.

Compared with the gemcitabine‐sensitive PDAC samples, the gemcitabine‐resistant PDAC samples achieved higher enrichment scores in the DDR and metabolic pathways and lower enrichment scores in the immune pathways. As reported by Jain *et al*. [50], gemcitabine‐resistant PDAC showed up‐regulation in glycolysis, pentose phosphate pathway, fatty acid synthesis and purine/pyrimidine synthesis. And, up‐regulation of glycolysis could maintain the EMT phenotype and reduce responsiveness to the therapeutic agent for PADC cells [51]. In addition, the activation of DDR pathways may counteract toxic effects induced by gemcitabine [50]. For example, the *ERCC1* gene is involved in multiple DDR pathways and overexpression of *ERCC1* is well documented in poor gemcitabine responders [52]. Delvecchio *et al*. [53] observed that the combination of gemcitabine and chemokine (*CXCL13* and *CCL21*) could potentiate antitumour activity of chemotherapy and increase the infiltration of CD8^+^ T cells. Xiao *et al*. [54] found that the high‐risk group with gemcitabine resistance showed increased macrophages M0 infiltration and decreased CD8^+^ T‐cell infiltration. Indeed, in our results, the sensitive PDAC samples achieved high enrichment scores in the chemokine signalling pathway and consistently high infiltration proportion in CD8^+^ T cells.

In single‐cell analysis, cancer cells in the same PDAC sample had both resistant and sensitive cancer cells, which could be used to explain why PDAC patients who responded to gemcitabine at the beginning will develop secondary resistance later. Zou *et al*. [55] also proposed complete responses to drug therapies are rare in tumours, and only some but not all subpopulations in a given tumour response to therapy. Single‐cell analysis revealed that most genes in the 28‐GPS were not only expressed in cancer cells but also in stromal cells. Thus, the 28‐GPS may represent a tumour stromal component and could be considered as a dictate of response to gemcitabine. In addition, some carcinogenic genes or pathways, such as *ANGPTL4* gene and TGFβ signalling pathway, were reflected in cell communication of the resistant group. *ANGPTL4* has been found to play an important role in the process of tumour metastasis [56]. The activation of TGFβ signalling pathway could promote resistance to gemcitabine in PDAC cells in a coculture assay *in vitro* [57].

Although our work was limited by the independent PDAC data sets with gemcitabine information to investigate the robustness of 28‐GPS, we used PDAC samples with prognostic information and PDAC cell lines with gemcitabine‐used information to validate the 28‐GPS. In addition, resistant samples in our work showed high metabolic levels and high‐fidelity DNA damage repair, which warrants our future detailed biological experiments to validate those discoveries.

## 5 Conclusions

In summary, we developed the 28‐GPS for gemcitabine based on transcriptome, which could be applied to predict response to gemcitabine chemotherapy for PDAC. The resistant samples classified by 28‐GPS in TCGA showed multidimensional resistance‐related characteristics compared with the sensitive samples. Collectively, it is worthwhile to further evaluate the clinical applications of 28‐GPS, which may assist clinicians to make a suitable strategy for PDAC patients.

## Conflict of interest

The authors declare no conflict of interest.

## Author contributions

YYG and KDL conceived the research; KDL, YDG and LZW performed the data processing and analysis; HHX, MZ and YWL carried out the data collection; KDL wrote the manuscript; YYG, ZXZ and TTC revised the manuscript; SLW, LS and FYX provided valuable suggestions for this manuscript; All authors have approved the final manuscript.

## Supporting information


**Fig. S1.** The workflow of this study.
**Fig. S2.** The genomic landscape between gemcitabine‐resistant and ‐sensitive samples classified by 28‐GPS in TCGA.
**Fig. S3.** The differences in immune infiltration assessed by five algorithms.
**Fig. S4.** Single‐cell annotation and copy number variation.
**Table S1.** The PDAC cell lines used in this study.
**Table S2.** The pharmacological data sets used in this study.
**Table S3.** The composition of 28‐GPS.

## Data Availability

The authors declare that all data supporting the findings of this study are available from public databases. The detailed information of these data sets is stored in Table 1 and Tables S1 and S2.
