# Supplementary material for: Systematic exploration of the underlying mechanism of gemcitabine resistance in pancreatic adenocarcinoma
Source: Mol Oncol. 2022 Jul 22;16(16):3034–51. doi: 10.1002/1878-0261.13279 (PMC9394232; doi:10.1002/1878-0261.13279)

**Supplementary Table 1**. The PDAC cell lines used in this study.

| Data | Cell line | PDAC  Cell line | Gene | Data source |
| --- | --- | --- | --- | --- |
| CCLE | 1376 | 49 | 19177 | https://depmap.org/portal |

**Supplementary Table 2**. The pharmacological datasets used in this study.

| Data | Cell line  source | Sensitivity  measure | Cell lines | PDAC cell lines | Drug | Data source |
| --- | --- | --- | --- | --- | --- | --- |
| CTRP | CCLE | AUC | 645 | 35 | 481 | https://ctd2-data.nci.nih.gov/Public/Broad |
| CGP | CCLE | IC50 | 639 | 17 | 130 | https://www.ncbi.nlm.nih.gov/pmc/articles/PMC3349233/ |
| GDSC1 | CCLE | AUC | 987 | 30 | 345 | https://www.cancerrxgene.org |
| GDSC2 | CCLE | AUC | 809 | 29 | 175 | https://www.cancerrxgene.org |

**Supplementary Table 3**. The composition of 28-GPS.

| Gene1 | Gene2 | *P* value | Gene1 | Gene2 | *P* value |
| --- | --- | --- | --- | --- | --- |
| FAM83A | LPXN | 1.91e-2 | AHSG | RPGRIP1 | 3.26e-2 |
| FAM83A | IFFO1 | 9.21e-3 | DSG3 | CLCN2 | 8.21e-3 |
| FAM83A | DISP1 | 1.69e-2 | CDH3 | CYP27A1 | 6.11e-3 |
| ERCC6L | ABCB4 | 3.70e-2 | NXNL2 | RPGRIP1 | 8.87e-3 |
| CDH3 | CCND2 | 2.15e-3 | FAM83A | GK5 | 1.82e-2 |
| MFN1 | RC3H1 | 8.87e-3 | CEP55 | TATDN3 | 1.69e-2 |
| FAM83A | LAT2 | 1.69e-3 | DSG3 | GBGT1 | 4.81e-4 |
| CEP55 | LSM6 | 3.70e-2 | DSG3 | TATDN3 | 1.39e-4 |
| FAM83A | MCOLN1 | 1.82e-2 | DSG3 | ZMYND15 | 6.35e-4 |
| FAM83A | IL27RA | 3.76e-3 | BUB1 | TATDN3 | 1.91e-2 |
| SENP2 | TBXAS1 | 3.26e-2 | SLCO1B3 | BRSK1 | 2.31e-2 |
| FAM83A | TTC13 | 1.91e-2 | MBOAT2 | ITGAM | 1.50e-2 |
| DSG3 | DISP1 | 1.39e-4 | CDH3 | GNPTG | 4.79e-3 |
| NXNL2 | C6orf58 | 2.51e-2 | CYB561 | MAN2B1 | 8.21e-3 |

**Supplementary Figure 1.** The workflow of this study. (**A**) Identifying gemcitabine resistant and sensitive samples in TCGA. (**B**) Development of gemcitabine signature based on REO patterns. (**C**) Validation of gene pairs signature in the independent cohorts. (**D**) Exploring the underlying gemcitabine resistance mechanism.


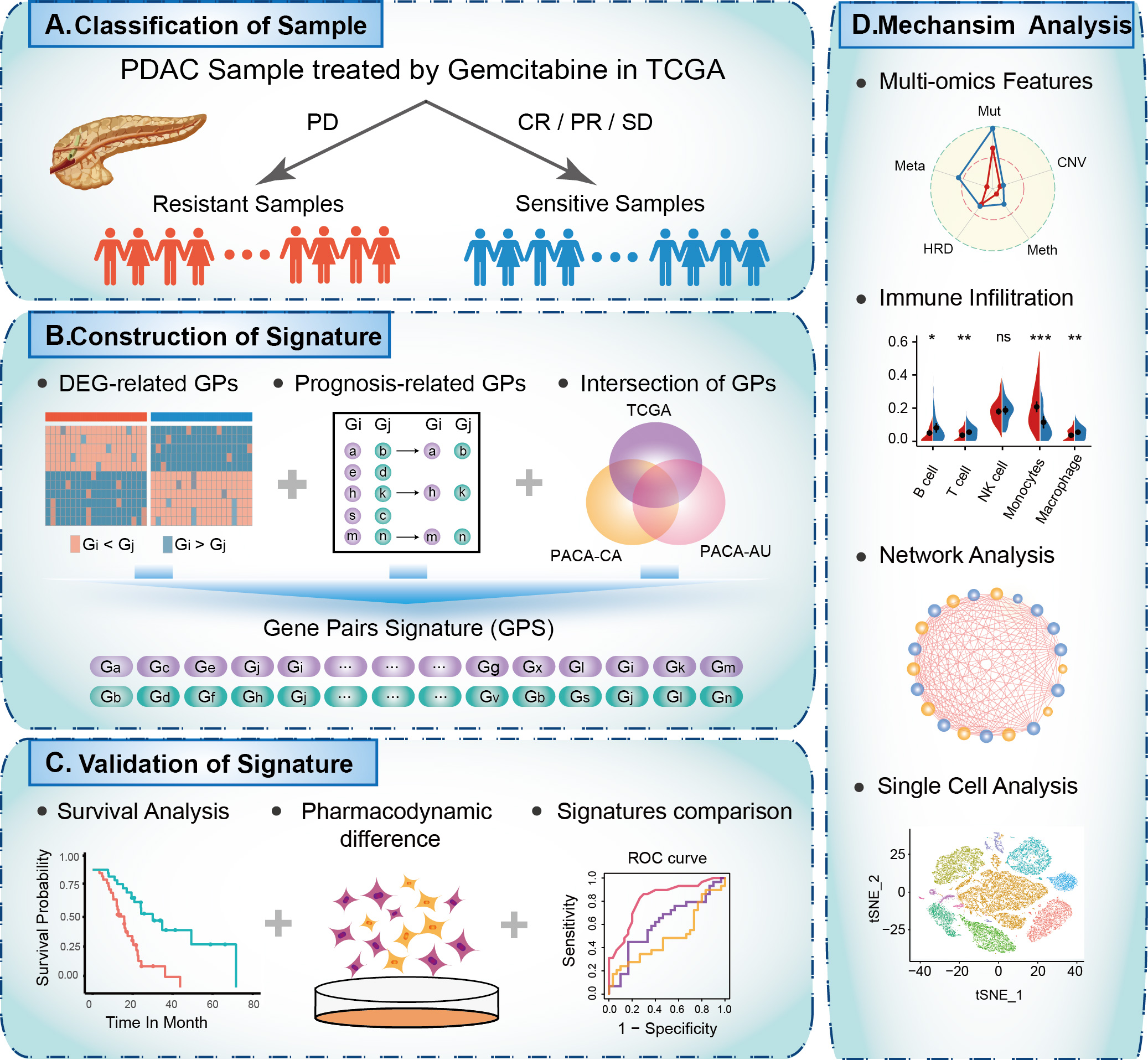


**Supplementary Figure 2.** The genomic landscape between gemcitabine resistant and sensitive samples classified by 28-GPS in TCGA. (**A**-**B**) The overlap of the 28-GPS, 14-gene signature and Gempred signature at the gene level (**A**) and pathway level (**B**). (**C-E**) 28-GPS resistance scores of different somatic mutations of *TP53*, *SMAD4* and *CDKN2A*. (**F**) The percent of amplification and deletion difference of CNV between gemcitabine resistant and sensitive samples. (**G-H**) Venn map shows the intersection of under-expressed and hypermethylated genes (**G**) and over-expressed and hypomethylated genes (**H**).

**
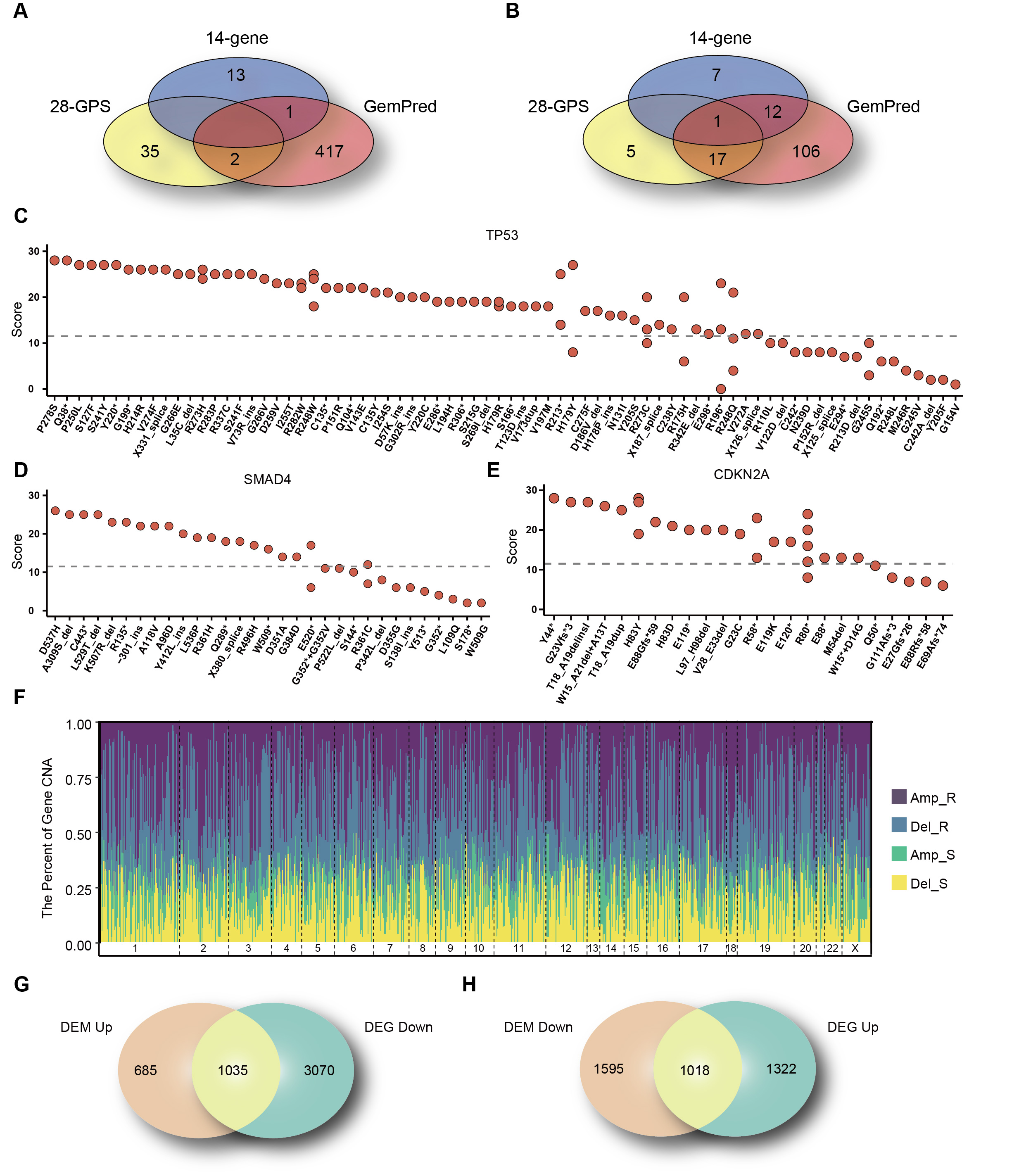
**

**Supplementary Figure 3.** The differences in immune infiltration assessed by five algorithms. (**A**-**E**) The proportion of immune cell infiltration between gemcitabine resistant and sensitive samples classified by 28-GPS in CIBERSORTx (**A**), EPIC (**B**), TIMER (**C**), QUANTISEQ (**D**) and XCELL (**E**). (**F**) The percent of immune cell infiltration between gemcitabine resistant and sensitive groups in five algorithms. The red arrows represent inconsistent infiltration proportion.


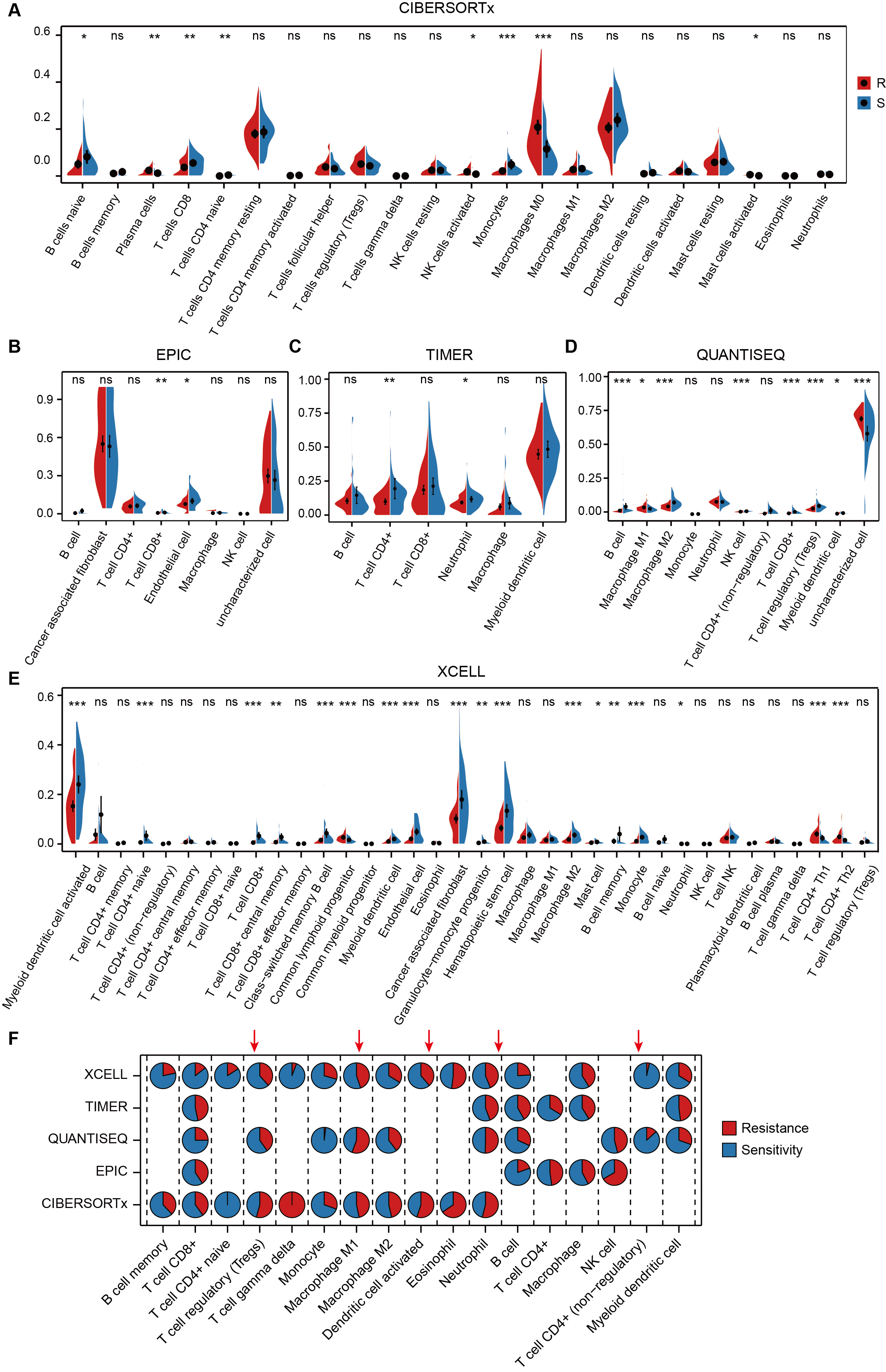


**Supplementary Figure 4.** Single cell annotation and copy number variation. (**A**) Bubble plot shows the expression pattern of canonical cell marker genes, with cell types in rows and genes in columns. The size of each bubble represents the fraction of cells with expressed corresponding genes and color represents the level of gene expression. (**B**) Violin plot shows the CNV score of different cell types in 24 PDAC samples.


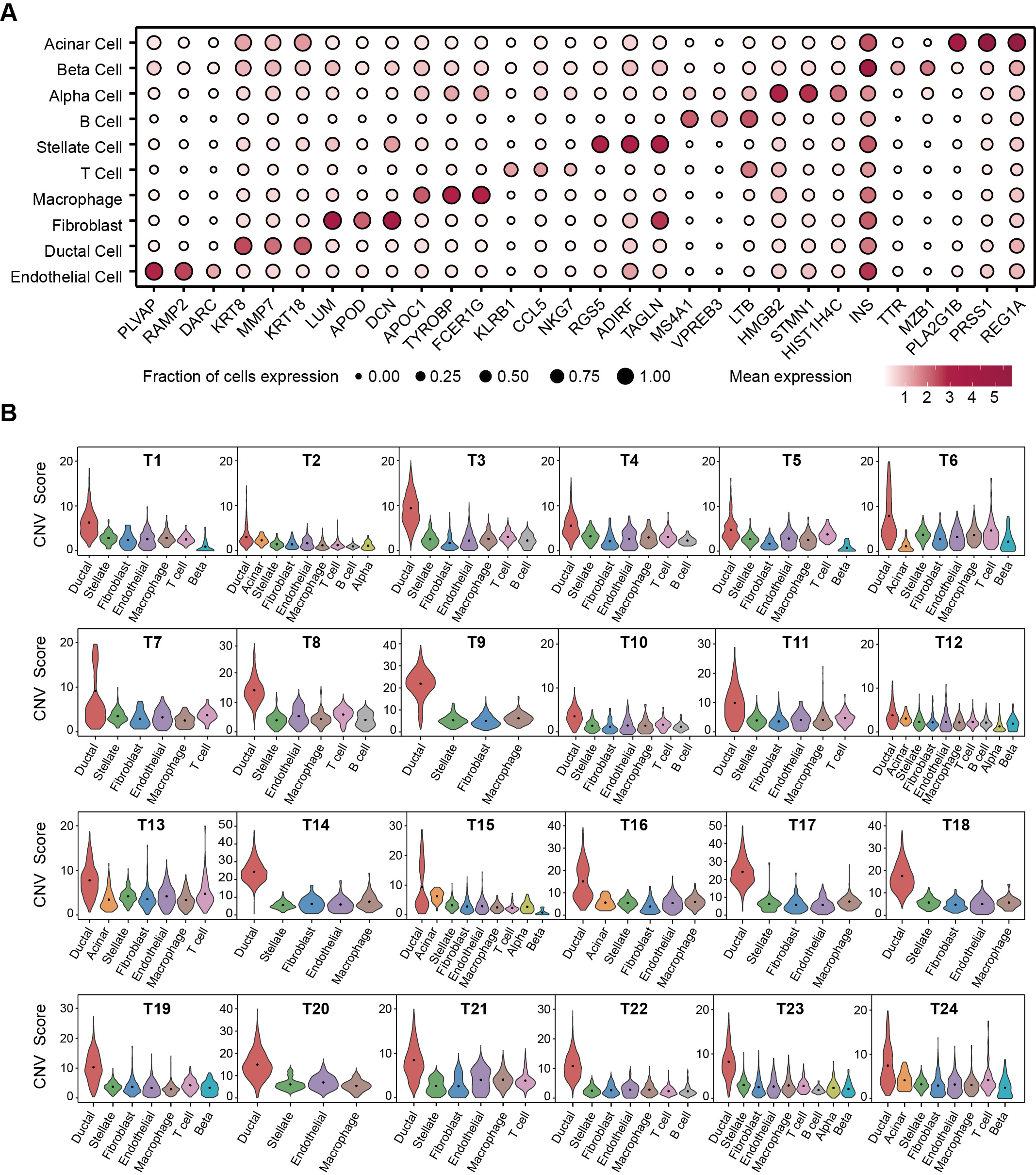

Supplement: Supplementary file 1 — Fig. S1. The workflow of this study. Fig. S2. The genomic landscape between gemcitabine‐resistant and ‐sensitive samples classified by 28‐GPS in TCGA. Fig. S3. The differences in immune infiltration assessed by five algorithms. Fig. S4. Single‐cell annotation and copy number variation. Table S1. The PDAC cell lines used in this study. Table S2. The pharmacological data sets used in this study. Table S3. The composition of 28‐GPS. [file MOL2-16-3034-s001.doc]
